# "One country, two systems": Sociopolitical implications for female migrant sex workers in Hong Kong

**DOI:** 10.1186/1472-698X-8-13

**Published:** 2008-12-12

**Authors:** William CW Wong, Eleanor Holroyd, Emily Y Chan, Sian Griffiths, Amie Bingham

**Affiliations:** 1 Department of General Practice, University of Melbourne, 200 Berkeley Street, Carlton, Victoria, 3053, Australia; 2 Division of Nursing and Midwifery, School of Health Sciences, RMIT University, Bundoora, Melbourne, Australia; 3 School of Public Health, The Chinese University of Hong Kong, Prince of Wales Hospital, Shatin, Hong Kong

## Abstract

**Background:**

Under the "two countries, one system" policy implemented by China to manage the return of Hong Kong's sovereignty, Hong Kong has maintained a comparatively prosperous economy within the Asian region. This has resulted in an environment which fosters migration from the mainland to Hong Kong, due largely to proximity, higher earning potential, common language, and a relaxing of border control measures. However not all mainland China citizens are equally able to access these new migration schemes and indeed a number of women such as sex workers are either migrating and/or working illegally and without occupational, legal and health protection within Hong Kong.

**Discussion:**

Female migrant sex workers are exposed to a number of significant threats to their health, however their illegal status contributes to even greater vulnerability. The prevailing discourses which view these women as either "trafficked women" or as "illegal immigrants" do not adequately account for the complex situations which result in such women's employment in Hong Kong's sex industry. Rather, their position can best be understood within the broader frameworks provided by migration literature and the concept of "structural violence". This allows for a greater understanding of the socio-political issues which are systematically denying migrant sex workers adequate access to health care and other opportunities for social advancement. When these issues are taken into account, it becomes clear that the current relevant legislation regarding both immigration and sex work is perpetuating the marginalised and vulnerable status of migrant sex workers. Unless changes are made, structural barriers will remain in place which impede the ability of migrant sex workers to manage their own health needs and status.

**Conclusion:**

Female migrant sex workers in Hong Kong are extremely vulnerable to a number of occupational health and safety hazards which have significantly detrimental effects on their health. These risks can best be understood within a broad framework of socio-political factors contributing to their vulnerability. Ensuring that migrant sex workers have adequate support for their health and legal rights requires require structural interventions such as decriminalisation and providing open and inclusive access to health service to counteract such factors.

## Background

"One country, two systems" was an innovative political experiment, proposed by the former Paramount Leader of China, Deng Xiaoping, in the 1980s. In essence, it was a framework for the governance of a unified China, allowing regions such as Hong Kong to maintain a high degree of autonomy with respect to political, legal and health systems, whilst deferring to the Chinese government on broader regional and global issues such as foreign policy and defence. Under this framework, Hong Kong has been able to maintain the capitalistic and democratic core of its society, whilst allowing mainland China to preserve its claims to its socialist roots. Though a part of China, Hong Kong remains ideologically separated, with clearly defined borders with China and the rest of the world. At the same time, the manner in which Hong Kong participates in and contributes to international issues is directly mediated by the policies of China.

In the following paper, we examine the broad socio-political environment that is fostered by the "one country, two systems" policies, and some of the unintended consequences in respect to migrant women. We suggest that these socio-political structures represent a form of "structural violence" against particular sectors of society, undermining a vulnerable sub-population's capacity for self-determination with resultant detrimental effects on access to healthcare and social agency. Taking female migrant sex workers (FMSWs) in Hong Kong as an example of a marginalised and vulnerable population, we argue that the current policies both contribute to and perpetuate their disadvantaged status. We suggest that the dominant discourses which seek to focus on migrant female sex workers as either victims of trafficking or as transgressors of immigration laws do not sufficiently capture the complexities of their situation, as they focus almost entirely on the role of an individual's agency. Our perspective is that the prevailing public dominance of these discourses allows the governments of both Hong Kong and China to abrogate their responsibilities to the health and welfare of this sub population.

The situation of migrant sex workers may best be understood not only within the discourses listed above but within the broader context of migration discourse, which offers more scope for taking account of the role of socio-political structures in influencing how migrant sex workers capacity for self-determination may be enacted. In doing so, we suggest the broader socio-political environment arising from the "one country, two systems" approach is systematically denying these women the right to social and economic progression, whilst failing to afford them basic rights such as access to health services and protection from exploitation and abuse. To conclude we propose policy and healthcare practise based interventions that may help to rectify these concerns.

## Discussion

### Sex work in Hong Kong

Although often considered too expensive a city to be a genuine destination for international sex tourists, Hong Kong has a thriving local sex industry [1]. Though no reliable data exists pertaining to the number of migrant sex workers, some estimates have placed the total number of female sex workers in Hong Kong at around 200,000 within a population of 6.8 million people [2]. Around 94% of women arrested for prostitution, however, are from mainland China [1]. The conditions for sex workers in Hong Kong are far from adequate. For a variety of reasons, many FMSW feel that they cannot report crimes committed against them, and as such become open to abuse and exploitation. Though sex work is itself legal in Hong Kong, most related activities are not, including soliciting for an immoral purpose, and loitering for the purpose of soliciting (Crimes Ordinance, s 147), advertising prostitution (Crimes Ordinance, s 147A) and running a vice establishment, which includes the use of a premise by more than one person for prostitution (Crimes Ordinance, s 137) [3].

In recent years there have been a growing number of reports of harassment of sex workers by police. In one of the few studies of FMSW in Hong Kong, some women claimed that they had been arrested for soliciting, despite the fact that the policemen had initially approached and engaged them [1]: at one stage, a sex worker claimed in her suicide note that she had been the subject of similar harassment [4]. At times, the police themselves have been guilty of subjecting the women to public humiliation and degradation, as in the instance when a number of sex workers were arrested and held in a "cage", situated in a public care park and able to be seen by the public [5,6]. Sex workers also reported instances of being strip-searched, slapped, and pressurised into signing confessions [7]. By reporting crimes to the police, the women identify themselves as sex workers, raising the possibility that they will be subject to future harassment or deportation.

In addition, these FMSWs – largely from mainland China – are doubly discouraged from reporting abuses because they are working illegally in Hong Kong. Many enter Hong Kong on temporary visas, the "Two-Way Permit" or on a visitor's visa, neither of which allow the holder to undertake any form of employment (including sex work). If the holder engages in work, they can be arrested for "breach of condition of stay" (Immigration Ordinance, s 41). Alternatively, those who enter without the appropriate documents (undocumented or illegal entrants) can be charged with remaining in Hong Kong without authority (Immigration Ordinance, s 38) [8]. In reporting abuses against themselves, FMSWs draw attention to themselves, generating the possibility that they will be punished for the above crimes. The punishments for such crimes are severe, and include heavy fines, imprisonment ranging from three to fifteen months, and repatriation: for those arrested in police crackdowns for immigration offences, there is the possibility that they can be repatriated without trial, "blacklisted" and refused re-entry to Hong Kong [7]. The severity of these punishments, along with their public visibility, leave FMSWs vulnerable to abuse on a number of fronts.

In other cases, this abuse may take the form of harassment, physical abuse, rape or robbery of individual sex workers by their clients [9], and in others it may take the form of more systematic abuse from those wishing to profit from the sex industry. This can include deceiving women into working in the sex industry [1], agents and pimps refusing to allow women to leave their dwellings when not working, forcing them to pay off enormous "agency fees", not allowing them to refuse clients, even when those clients will not wear a condom, and forcing them to work an exorbitant number of hours and days in a week [10].

The prevailing conservative attitudes in Hong Kong also mean that FMSWs are highly socially stigmatised, in light of both their status as migrants and their role as sex workers [9,11]. Such stigma not only contributes to poor health directly, through the impact that it has on a person's psychological and emotional well-being, but also in the sense that, once again, it prevents people from taking action which may potentially "out" them as a sex worker: such as actively seeking health care for STIs, or seeking protection from crimes committed against them. And, finally, risks to the health of FMSWs are compounded by the fact that, as non-residents of Hong Kong, they are charged a high fee when accessing health services – well beyond that which most can afford, meaning that many do not seek medical assistance, even when treatment is needed [12]. For a number of FMSWs, these issues can be exacerbated by the fact that many are illiterate and have no knowledge of HIV and STIs, and therefore lack the knowledge required adequately to protect themselves [11].

### Migrant sex workers: Powerless or Empowered?

FMSWs- in Hong Kong and elsewhere- are often conceived of as being the victims of "trafficking", a highly emotional and politicised terms that is becoming increasingly problematic. Though countless definitions of trafficking exist, most share basic similarities, and refer to compelling people, through *force*, deception or coercion, into a situation in which their free will is denied, in tandem with the pursuit of commercial profit [e.g. [13-15]]. The idea that women must be forced into sex work sits easily in the public and policy imagination of what sex work entails, and with what is known (and surmised) about the conditions in which it is undertaken. Some influential trafficking literature of recent years has come to focus on the "victimhood" of the women, highlighting the role of those who orchestrate and facilitate the machinations of trafficking. This tendency can be found within the United Nations Convention against Transnational Organised Crime (UNCOTC, 2000) [16], under the supplementary Protocol to Prevent, Suppress and Punish Trafficking in Persons, Especially Women and Children ("Trafficking Protocol") and the Protocol against the Smuggling of Migrants by Land, Sea and Air ("Smuggling Protocol") [15,17]. Under the Trafficking Protocol, a woman is considered to be trafficked if she is employed in exploitative conditions, regardless of her consent to those conditions, and certain rights are owed to trafficked people (health services, legal rights, housing, etc.) [14]. Within the discourse of trafficking, women are often seen to be passive, and in need of "rescue and repatriation", that is, that the best thing for them is to be dealt with humanely in their destination country/region, then removed from the situation into which they have been trafficked, and returned "home" [17].

By contrast, the bureaucratic discourse which focuses attention on the legal status of sex workers completely bypasses the circumstances and motivations of individuals, replacing it instead with the rights of the state. This is the discourse within which the Hong Kong government tends to operate. Since the loosening of visa restrictions on travel between Hong Kong and China in recent years, the rate at which mainland women are arrested for immigration offences in Hong Kong has reached enormous proportions, climbing from 3646 in 2000 to 11794 in 2005, representing approximately 75% of the female inmate population [1]. A "significant portion" of women incarcerated have been found to be sex workers [18]. This proportion has been estimated to be as high as 40% [19,20]. When applied strictly, laws such as the immigration laws of Hong Kong imply that a "black and white" situation exists in which no extenuating circumstances exist which may "justify" a woman's illegal migration into Hong Kong or the illegal pursuit of paid employment. In effect, it assumes that all women are capable of free choice when it comes to migration and employment, suggesting that those who break the law have done so willingly and freely, and can therefore be punished appropriately.

### Structural Violence

While the above discursive frameworks no doubt capture some aspects of the complexity surrounding FMSWs rights to health and legal protection, we contend that for the most part, they are oversimplifications. Within them, it is possible to make a clear distinction between "willing" and "unwilling" ("unforced" versus "forced") migration, which guide subsequent attempts to aid or punish the migrants accordingly. In truth, however, simply assuming that trafficked women are purely passive victims who need and want to go "home" ignores the fact that there may truly be greater opportunities for economic and social advancement in their destination countries that trafficked women wish to pursue. Even while wanting to be released from debt bondage and unreasonable working conditions, women may wish (in the name of gaining economic independence and security, the ability to support children, and so on) to remain in the country to which they were "trafficked" [17]. Similarly, assuming that "illegal immigrants" were able to make an entirely free choice when deciding to migrate and work illegally denies that the choices a woman has can be severely restricted by structural (social, political, economic etc.) factors beyond their control.

Instead, by viewing FMSWs in Hong Kong within the context of migration, and economic migration in particular, the broader socio-economic environment in which mainland women's migration to Hong Kong takes place is brought to the fore. Theories of economic migration see migration as a reaction to labour market and economic incentives [21]. However, within the migration literature, the line between "willing" forms of migration ("economic migration") and "forced" migration has become blurred [22], with many apparently willing migrants *compelled *to do so by unfavourable circumstances in their home regions (such as a lack of viable economic opportunity).

The concept of "structural violence" fits well within this discursive context. The medical anthropologist Paul Farmer contends that the comparatively poor status of vulnerable and marginalised people is a result of structural violence: neither culture nor pure individual will is at fault; rather, historically given (and often economically driven) processes and forces conspire to constrain individual agency. Structural violence is visited upon all those whose social status denies them access to the fruits of scientific and social progress." [23]. We believe that such a situation has been fostered in the Hong Kong-China socio-political climate by the "one country, two systems" policies.

Hong Kong's autonomy and the capitalistic framework allowed by this relative independence under the 'two systems' policies have allowed its economy to flourish, particularly when compared to others within the region. It is under these terms, however, that the conditions have been generated which make migration from China to Hong Kong (whether willing, unwilling, and everything in between) both desirable and feasible. For women in China, for example, particularly those from rural areas, the opportunities for economic and social advancement are limited. Gender inequality is rife, with few employment prospects for women, and wages lower than those available to men. [24]. Migration in search of employment is common in China, particularly from rural to urban regions, and it is estimated that up to a quarter- or even a third – of the population in China's urban centres are migrant workers [25].

As with China's urban centres, Hong Kong presents an attractive destination for workers: thanks largely to its capitalist economy it remains a comparatively wealthy city within the region, and the opportunities exist for migrating women to make more money than would be possible on the Chinese mainland. In addition, with closer political ties under the "one country" agreement, Hong Kong has relaxed a number of restrictions on mainland Chinese citizens travelling to Hong Kong – largely in search of tourist dollars – making cross-border migration an easier proposition [9]. Migration in search of employment then, is both well established as a viable option in China- including the option of undertaking sex work- and Hong Kong has been established as an attractive and feasible destination, thanks to an established sex industry, higher potential earning capability and easing of travel restrictions. Under conditions such as these, the idea that illegal immigrants migrate "voluntarily" becomes problematic. The socio-political situation in China means that many women must migrate to find employment, due to factors that are largely out of their control but which depend upon the actions and choices of their government. Interviews carried out with FMSWs who had been arrested for immigration/soliciting offences have indeed indicated that "financial considerations" – either fleeing from "urgent and dire economic circumstances" or pursuing higher earnings than were available in the factories and service industry in China – was the primary factor influencing many women to enter Hong Kong [1].

By focussing attention on the bureaucratic status of FMSWs working in Hong Kong (and the "voluntary" nature of their migration), the government of Hong Kong is effectively able to elide responsibility for fostering the structural issues which generate the need for FMSW to migrate. At the same time, the fact that Hong Kong follows the lead of China on international issues further exacerbates this problem: though having ratified the UNCOTC, China is neither a signatory to the Trafficking Protocol nor the Smuggling Protocol. Neither China, nor by default Hong Kong are therefore bound by these protocols which recognise the vulnerable status of these women, or actively punish those who take advantage of them by abusing their vulnerable status. This is despite credible reports emerging which suggest trafficking does occur in Hong Kong [14]: these include the discovery of two women huddled in a phone booth in Hong Kong, claiming to be victims of a trafficking scheme [26].

In attempting to set out an agenda for tackling the issue of trafficking, the United States State Department have developed a system of "ranking" countries that they have identified as having a trafficking problem: Hong Kong has consistently been ranked as a Tier 1 country, meaning that they have "fully complied with the minimum standards" set out to combat trafficking in the United States Victims of Trafficking and Violence Protection Act of 2000 [14,27], having trained officers to identify trafficked women, offering legal protection to trafficked women, among other initiatives. However, it is tempting to view these claims as "lip service": in the interviews conducted by Laidler et al. (2007) with detained FMSWs, twelve women indicated that they had been deceived and coerced into sex work, receiving little response when they informed the authorities of this [1]. For the year preceding the publication of the 2008 Trafficking in Person's Report, Hong Kong authorities recorded only two convictions for trafficking, and two other reports of trafficking for prostitution, and no laws exist that specifically target traffickers [28]. This is in stark contrast to the large number of women who are arrested for immigration offences every year.

### Implications and the way forward

We have argued above that a combination of structural factors, illustrated by the "one country, two systems" policies in Hong Kong and China, have resulted in FMSWs residing in Hong Kong being extremely vulnerable to abuse and ill-health, and that within the current socio-political environment found in Hong Kong and China, they are given little opportunity for self-determination or for any course of action which will allow them to improve their social and economic standing. We believe that protection from abuse and access to health services are fundamental human rights, and that dealing adequately with global health inequalities requires a commitment to global human rights, and vice versa. In the case of FMSWs in Hong Kong, and in other neighbouring Asian countries with similar push pull factors, we suggest that structural forces have combined to disempower and marginalise such women and, as such, require structural interventions to reverse this trend. These interventions must provide sex workers with genuine alternative forms of employment, without negatively impacting upon the rights of those who survive by working in the sex industry [29]

One major contribution that the Hong Kong and Chinese authorities could make to protecting FMSWs from various forms of abuse would be to become signatories to both the Trafficking Protocol and the Smuggling Protocol. Though the gap between policy and practice is sometimes large; if China were to agree to the protocols, then even if active enforcement of those protocols did not follow, sex workers would at least have an additional legal avenue through which they could pursue their individual claims to justice. In addition, it would send a strong public message in support of trafficked women who find themselves forced into sex work, which would help to generate a more favourable, less stigmatising perception of such women.

Another plausible option is the decriminalisation of sex work and related activities. Decriminalisation will allow for the regulation of sex workers' industrial conditions and occupational health and safety [30]; increased capacity to seek police protection against abuse, without fear of criminalisation [31]; decrease police harassment; and increase their visibility and accessibility for health promotion efforts [32]. Another point at which decriminalisation could occur is in the relaxing of immigration laws, by creating visas which allow migrants legally to engage in sex work: illegal immigrant status forces sex workers "underground", again resulting in poor access to health services, discrimination, violence, STI or HIV acquisition and exploitation [33].

In addition, adequate health and social care assistance must be provided to FMSWs so that they are best able to protect their health. Ideally, this would take the form of granting economically viable access to health services. However, given the current status of FMSWs as illegal migrants, this is problematic (particularly as other non-residents are not currently granted such access). More appropriate perhaps would be an expansion in the role of non-governmental organisations (NGOs) who currently seek to provide FMSWs in Hong Kong with supplies such as condoms, health education, information about their legal rights and assistance with legal aid, counselling, and various other methods aimed at fostering and enhancing a sense of empowerment and social agency. Examples of such organisations currently in operation include Action for REACH OUT (AFRO) [34] and Zi Teng [35]. Though these organisations do an excellent job, our personal experience in working with FMSWs in Hong Kong and the region lead us to believe that an expansion of such efforts is required. Again, government support for such initiatives need not take the form of direct funding, but could instead be targeted at increasing the authorities' understanding of the need for such services, the complexity of issues involved, and the potential benefits that such programs may have for the FMSWs and the public.

In 1986, the Ottawa Charter described Health Promotion as "the process of enabling people to increase control over, and to improve, their health" [36]. We believe that some or all of the initiatives mentioned above could be effective not only in addressing the immediate threats to the health of FMSWs in Hong Kong, but also for the long term safety and security of such women. Removing barriers which prevent FMSWs from being able to maintain or improve their health is a significant step towards providing them with a greater degree of social agency, and vice versa. Empowering FMSWs is an important aspect of health promotion, and of the related pursuit of ensuring their broader human rights.

## Conclusion

Hong Kong has a thriving local sex industry, with many sex workers having migrated from mainland China.

Migrant sex workers in Hong Kong are particularly vulnerable to stigmatisation, abuse and subsequent ill-health.

Dominant discourses of trafficking or "willing" migration do not adequately account for the complexity of circumstances leading to the migration of women for sex work in Hong Kong.

Current Hong Kong laws contribute to and perpetuate the marginalised and vulnerable status of migrant sex workers in Hong Kong.

Structural interventions are required to ensure that the rights of migrant sex workers in Hong Kong are not being compromised.

## Competing interests

WCW has the following conflicts of interest as the Ex-co member of Action for Reach Out, which is a non-governmental organization for the welfare and health of female sex workers in Hong Kong and medical consultant for Zi Teng.

EH was the Ex-Co member for Action for Reach Out and was Vice President between 2003–6.

There is no funding support for this study.

## Authors' contributions

William CW Wong participated in the design and write up of the main text of the report. He has seen and approved the final version. EH discussed the concept of this paper, helped to formulate and write up the section on ways forward as well as commenting on the rest of the manuscript. EYC conducted some literature searches, provided information on international data on the discussed topic and commented on the final draft. SG edited and provided substantial advice on this paper. AB participated in the literature search and with the structuring and write-up of the final report.

## Pre-publication history

The pre-publication history for this paper can be accessed here:


